# Optimal Tilt Angle Determination for PV Panels Using Real Time Data Acquisition

**DOI:** 10.1002/gch2.201900109

**Published:** 2020-02-18

**Authors:** Manoj Kumar Sharma, Deepak Kumar, Sandeep Dhundhara, Dipesh Gaur, Yajvender Pal Verma

**Affiliations:** ^1^ Department of EEE University Institute of Engineering and Technology Panjab University Chandigarh 160014 India; ^2^ Department of Basic Engineering College of Agricultural Engineering and Technology CCS Haryana Agricultural University Hisar Haryana 125004 India

**Keywords:** Chandigarh, optimal tilt angle, real time, renewable energy, solar panels

## Abstract

Solar energy is one of the promising renewable energy sources which has the potential to meet the future energy demand around the world. To maximize the irradiance fall, solar panels are generally equipped with a motor tracking system and are placed at a specific tilt angle. However, tracking methods are not cost‐effective and a fixed tilt angle is not productive. This study proposes a method for harnessing maximum output from photovoltaic (PV) panels throughout the year by determining the optimal tilt angle. The investigation is performed on real‐time solar PV panels of 5 kWp rated capacity installed at 10°, 20°, 25°, 30°, and 40° angle on the rooftop of engineering institute situated at Chandigarh, India. The real‐time power generation response for a year is used to find the optimal tilt angle. The results obtained from the practical setup are validated by comparing it with the simulation results of the regression analysis. In addition, the impact of the optimal angle on total power generation and carbon emissions is analyzed. The results reveal that the proposed approach is quite effective to increase the power generation of PV panels up to 7–8% and can be practically implemented in any location throughout the world.

## Graphical abstract

This study determines the optimal tilt angle of the PV panel for the Chandigarh region in India. The analysis reveals that total power generation through PV generating units is increased by 7–8% in a year by placing the PV panels at optimal angles compared to fixed angle at the studied location during different times of the year.

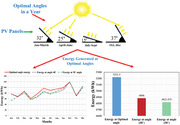

## 1 Introduction

**Figure 1:**
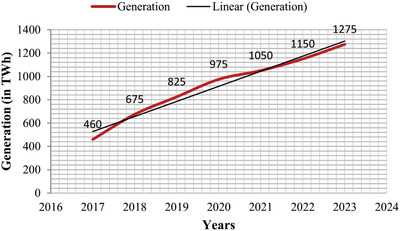
Estimation of solar PV generation around the world.[qv: 2]

Solar energy is inexhaustible and one of the cleanest renewable sources of energy. The solar power in the form of irradiance trapped by the earth is ≈1.8 × 10^11^ MW, which is far enough to solve all the present energy crisis in the world if it is used efficiently.[qv: 1] The power generation from solar photovoltaic (PV) has gradually increased all over the world in recent years. The global trend in solar power generation given by the international energy agency (IEA) ‘Renewables 2018' report from 2018 to 2023 is depicted in **Figure** **1**. It shows that the solar PV with 575 gigawatt (GW) capacity is expected to be operational in the world during the period.[qv: 2]

**Figure 2:**
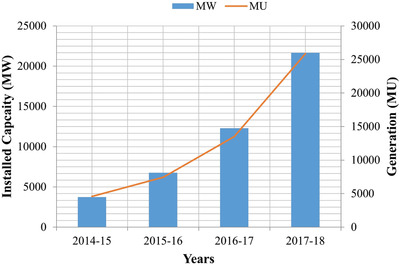
Solar installed capacity versus generation data.[qv: 3]

In India, the report of central electricity authority (CEA) mentioned that the government has fixed a goal for making renewable energy capacity of 175 GW by the year 2022.[qv: 3] The trend of solar capacity installed and generated in India for the previous 4 years is shown in **Figure** **2**.

Solar installed capacity for duration 2016–2017 is ≈13 500 MW which has been increased to 25 871 MW linearly and on generation hand, which has been increased from ≈12 289 MU to 21 652 million units (MU).[qv: 3] The ministry of new and renewable energy (MNRE) has identified 60 cities in India to be developed as ‘Model Solar city' as part of the national mission of solar energy with Chandigarh being one of them in the northern region. As of 2017, 16.20 MW of solar has already been installed with 9.40 MW being commissioned in the year 2016/17. In the master plan of ‘Chandigarh Solar city, it has a midterm target of 5 megawatt peak (MWp) rooftop solar by 2017 and a long term target of 10 MWp rooftop solar installation by 2022. As MNRE revised its target of 100 GW to be achieved by 2022, the revised target for Chandigarh administration is 100 MW solar PV by 2022.[qv: 4] The statistical analysis reveals that the modern world is emphasizing on the solar energy sector and solar energy future is very promising in India as well. Therefore, it is important to develop optimal harnessing methods for its proper usage. The efficient techniques to harness the PV energy is very crucial and the need of the hour.

Among various techniques of the effective harnessing of PV energy, the installation of solar panels at an optimal tilt angle can play an important role in order to enhance the generation efficiency of the PV‐based generating units. The generation efficiency of PV based generating units has mainly been affected by the amount of solar radiation incident on PV panels.[qv: 5] Solar radiation magnitude incident on panels depends on two important factors, direction and tilt angle of panels. The optimal tilt angle of the panel varies accordingly to the position of the sun with respect to the earth. It varies on a daily, monthly, and yearly basis. Also, the optimal angle depends upon the location. Therefore, it is very important to maintain an optimal tilt angle of the panel throughout the year to ensure maximum energy generation. For finding the optimal tilt angle for a particular area, its latitude, climate condition, solar radiation characteristics, and utilization period plays an important role.[qv: 5]

In the literature, various authors have proposed strategies for the effective harnessing of available solar energy for electricity generation. For finding optimal tilt angle, researchers have used different solar radiation models of the direct, diffuse, and reflective component of radiation, isotropic models[qv: 6–9] independent of atmospheric conditions), anisotropic models,[qv: 10–13] (depends on atmospheric conditions) or combination of both.[qv: 14,15] These radiations models are simulated mostly through MATLAB programming and the tilt angle has been optimized. The radiation data needed for the models are generally taken from National aeronautics and space administration (NASA) website or local meteorological department of the past years.[qv: 6,9,16,17] The real time data of radiation measured from the experimental setup has been used to validate the simulation results in ref. 18. **Table** **1** provides a brief introduction of the various studies performed by authors in order to investigate the optimal tilt angle as different locations. The methodology implemented, software used, and information about the data taken to find the optimal angle have been presented. There are two approaches developed by the researchers to calculate optimal tilt angle, one is an automatic solar tracking system and the other is manually set the angle for different time periods. The former approach used motors and other equipment which uses energy and are also expensive. Therefore, to calculate the optimal angle for a particular period, an effective approach and manually setting the angle for that period is required. Also, the surveyed literature reveals that most of the studies have been performed using software (i.e., MATLAB environment) via simulating solar radiation models.[qv: 9–12] Very few studies are available in which the hardware setup of a solar PV system has been used to find the optimal angle.

**Table 1:** Brief literature review regarding the studies executed by different authors to determine the optimal tilt angle of the PV

Author's Name and year	Methodology used	Software and data information	Location
Jacobson et al. (2018)[qv: 23]	PV watts calculator used to find solar panel output by varying tilt angle. Equation of optimal tilt angle as function of latitude is formed.	National renewable energy laboratory (NREL) PV watts calculator/global GATOR(Gas, Aerosol, Transport, Radiation)‐GCMOM(General‐Circulation, Mesoscale, and Ocean Model) meteorological station data	Major countries in the world
Ozbay et al. (2017)[qv: 18]	Manually setting of panel at different angles and input data to controller to calculate power and current output to find optimal tilt angle.	Raspberry pie microcontroller and python programming/real time data.	BIlicek city, Turkey
Kaddoura et al. (2016)[qv: 6]	MATLAB code has been made in which solar radiation data from NASA has been input which optimize tilt angle according to solar radiation.	MATLAB/NASA data	Different cities in Saudi Arabia
Morad et al. (2018)[qv: 10]	Bernard‐Menguy‐Schwartz model is used to made tilt angle model and programmed using the EES (engineering equation solver).	EES software used consist the programming structures of C and FORTRAN	Different cities in Iraq
Jamil et al. (2016)[qv: 7]	Liu and Jordan model used for total radiation estimation on inclined flat surface.	Pyranometers, shadow ring and SD data‐logger	Aligarh and New Delhi
Nfaoui et al. (2018)[qv: 16]	MATLAB code is used to estimate the totality of the solar radiation on any inclined surface, from which optimal angle under which the maximum energy could be absorbed by the solar cells has been determined.	MATLAB/ NASA data	Settat city in Morocco
Salari et al. (2017)[qv: 24]	MATLAB code has been developed to calculate the monthly average daily total solar radiation on a surface. The developed program calculates the optimum slope angles which correspond to the maximum amount of received irradiation.	MATLAB, Iranian Meteorological Organization total solar radiation data for the period of 1983–2012 are used.	Yazd, Iran
Salavati Pour et al. (2011)[qv: 14]	Three different mathematical models are used namely Liu, Klein, and Hay to determine the monthly optimal angle	Mathematical modelling, meteorological station data in Isfahan for eight years (2000–2008) using CM6B pyranometers.	Isfahan, Iran
Khatib et al. (2015)[qv: 8]	Liu and Jordan model for solar energy incident on a tilt surface have been used for finding the optimal tilt angle.	Mathematical modeling/real time data.	Different cities in Malaysia
Benghanem et al. (2011)[qv: 11]	Different isotropic and anisotropic mathematical models used.	MATLAB, NREL data (1998–2002) for Saudi station.	Madinah, Saudi Arabia
Asad Ullah et al. (2019)[qv: 25]	MATLAB code was developed to calculate the total solar radiation incident on a solar panel using Liu‐Jordan and Hay's model.	MATLAB, NREL, and ESMAP website past 17 years data for solar radiation.	Lahore, Pakistan
Dutta et al. (2016)[qv: 9]	Liu and Jordan model has been used to simulate solar radiation model.	FORTRAN computer program NREL and NASA data, pyranometer data used for validation.	Kolkata, India
Fung et al. (2005)[qv: 12]	Optimum angle has been calculated by using Hottel and Woertz and Reindl model to formulate solar radiation model.	On‐site measurement data has been used to validate the result of the simulation model.	Hong Kong
Shu et al. (2006)[qv: 26]	Small PV modules at different tilt angles have been installed and power output of modules has been determined and validated through solar radiation model. Second‐order polynomial equation has been deduced from experimental data to determine the optimal angle.	Experimental data from 4 modules (2002–2003) and radiation data from different weather stations, new energy and industrial technology development organization (NEDO).	Kitakyushu, Japan
W.G. Le Roux (2016)[qv: 27]	For each of the locations, the annual solar insolation on a fixed tilted surface was obtained for all the possible combinations of collector tilt angles. Validation is done through real time data.	SolTrace, a ray‐tracing software developed by the (NREL), real time data from SAURAN equipped with pyranometers and pyrheliometers	Different cities in South Africa
Yakup et al. (2001)[qv: 28]	A mathematical model was used for estimating the total (global) solar radiation on a tilted surface. Comparison between mathematical model and simulation has been done.	Meteorological Department, Ministry of Communications, Brunei Darussalam.	Brunei, Darussalam
Tang et al. (2004)[qv: 15]	Collares‐Pereira and Rabl model has been used for the estimation of diffuse‐radiation, Klein and Hamilton model used for *R*_b_ and *R*_d_	Mathematical modelling, Meteorological data.	Different cities in China
Tlijani et al. (2017)[qv: 29]	PV panel rotated at five different directions and angles.	MATLAB/SIMULINK/experimental data of two modules at different orientation/angles.	Tunisia
Sinha et al. (2016)[qv: 17]	HOMER software calculate global radiation on pv panel using Hay, Davies, Klucher, and Reindl model which is used to calculate the optimal tilt angle.	Hybrid optimization model for multiple energy resources (HOMER), Weathering station, NIT Hamirpur, NASA.	Different sites of Himachal Pradesh, India
Bailek et al. (2018)[qv: 13]	Perez9 model used in solar radiation mathematical model to determine optimal tilt angle.	MATLAB, local meteorological station data (Adrar station).	Adrar city, Algeria

Limited studies have been done in India with real time data on tilt angle determination of panels. Therefore, a maiden study has been performed using real time data collected from the rooftop PV panel to find the optimal tilt angle at the selected location (Chandigarh region). A detailed theoretical and practical analysis has been carried‐out to determine the optimal tilt angle. In addition, the experimental results have been validated using the regression technique. The main aim of the study is as follows:

- To statistically analyze the real time power output data for a year (every hour) of 5 kW solar panels placed at different angles.
- To determine the optimal tilt angles of the panels installed at 10°, 20°, 25°, 30°, and 40° degrees.
- To validate the experimentally calculated optimal angles using regression analysis.
- To investigate the impact of optimal tilt angle on emission reduction.
- To provide technical recommendations of the optimal tilt angle schedule of panels for the Chandigarh region on a quarterly basis which is feasible to implement.

## 2 Experimental Section

In this research work, the study of the optimal tilt angle of the PV panel for the Chandigarh region was carried out. The experimental setup was installed in University Institute of Engineering and Technology (UIET), Panjab University, Chandigarh. The whole setup comprised of three main components: i) PV panels, ii) micro‐inverters, iii) data acquisition system. These parts have been discussed briefly in the next sections.

### 2.1 PV Panels

**Figure 3:**
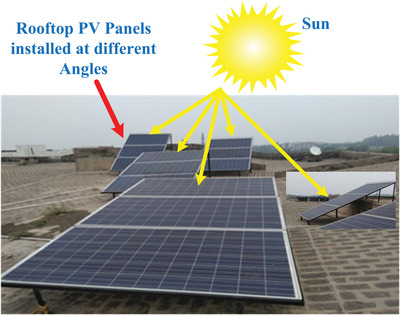
Solar PV modules installed on rooftop of the institute.

The setup consisted of five solar panels (Zytech Model ZT 320P) of 1 kW each rated capacity inclined at angle 10°, 20°, 25°, 30°, and 40° degrees on the rooftop of the institution building as shown in **Figure** **3**. Zytech polycrystalline PV modules are used in the present study. The detailed specifications of the module placed at five different angles are presented in **Table** **2**.

**Table 2:** Specification of ZT‐36V‐72C (320 Wp) PV module

Parameters	Rated capacity
Rated output current [A]	0.92 at 230 VAC
Rated voltage range [V]	180–260 VAC at 230 VAC
Static MPPT efficiency [%]	99.5 at 230 VAC
Maximum output efficiency [%]	97.0 at 230 VAC

The electrical power output (*P*_SPV_) of a solar PV panel can be expressed in (1):

(1) $$P_{\text{SPV}}\;\text{=}\;G_{\text{PV}}f_{\text{PV}}\left(\frac{I_{\text{T}}}{I_{\text{T},\text{STC}}}\right)\left[\text{1}\;\text{+}\;\alpha_{\text{P}}\left(T_{\text{C}}\text{-}T_{\text{C},\text{STC}}\right)\right]$$

where, *I*_T_ and *I*_T,STC_ denote the radiation (kW m^2^) incident on the PV panels at any instant of time and at standard test conditions (STC) (1 kW m^−2^), respectively. The rated capacity of the PV generators is denoted by *G*_PV_. *T*_C_ and *T*_C,STC_ denote the cell temperature (°C,) of the PV panels at any instant of time and at STC (25 °C), respectively. *f*_PV_ represents the derating factor PV system, α_P_ is the temperature coefficient of power (%/°C).[qv: 19]

Further, the velocity of wind and temperature around the cell of PV panels primarily impacted the output power of any PV panels. The energy balance corresponding to the PV system can be stated as given below:

(2) $$\left(\tau \alpha \right)I_{\text{T}}\;\text{=}\;I_{\text{T}}\eta_{\text{PV}}\;\text{+}\;U_{\text{L}}\left(T_{\text{C}}\text{-}T_{\text{a}}\right)$$

where, *τα* represents the actual transmittance–absorptance of the PV panel, η_PV_ and *U*_L_ denote the efficiency and surroundings heat transfer co‐efficient of the PV array, *T*_C_ and *T*_a_ designate the cell and ambient temperature of the PV module, respectively.[qv: 20] The value of η_PV_ is considered as zero at no‐load conditions.

### 2.2 Micro‐Inverter

**Figure 4:**
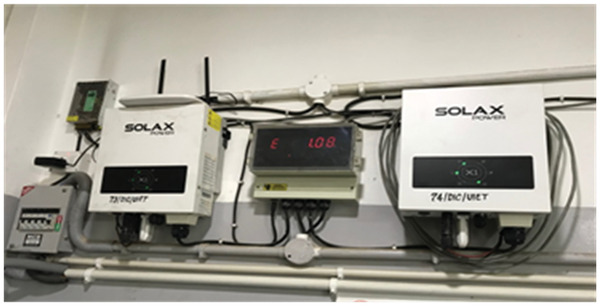
Micro‐inverter WVC 295.

The PV panels output was connected with the grid using grid‐tie micro‐inverters of SOLAX Pvt. Ltd. as shown in **Figure** **4**. Each PV module has its own grid‐tie micro‐inverter (WVC‐295) to change the DC into AC output. The real time data were collected and tracked through micro‐inverters and transmitted to the computer. The main specifications of the micro‐inverter are given in **Table** **3**.

**Table 3:** Specification of micro‐inverter WVC‐295

Technical specification	Value
PV maximum output voltage in 25 °C and 1000 W m^−2^ (V)	37.6
Maximum output current in 25 °C and 1000 W m^−2^ (A)	8.51
I_sc_ in 25 °C and 1000 W m^−2^ (A)	9.08
Output voltage in 25 °C and 1000 W m^−2^ (V)	46.2
Module efficiency	16.5%
Cell efficiency	18.6%
Operating temperature	−40 °C to 85 °C

### 2.3 Data Acquisition System

**Figure 5:**
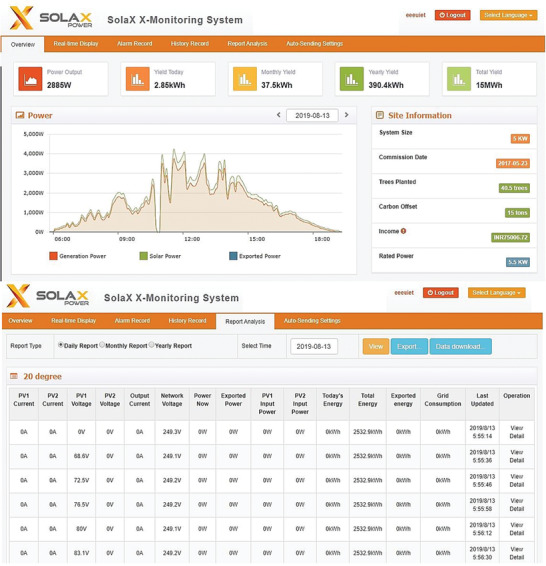
Solax real time monitoring portal.

SOLAX real time monitoring portal was used to display the micro‐inverter's data on the computer from anywhere through the internet. The electrical parameters output such as voltage, current, and power of the panel were monitored in real time from the system. The sampled data of PV panels of every 10 min came into the inverters and can be monitored on the portal. The real time data of PV panel generation and load power at different angles were displayed on the portal. The snapshots of SOLAX portal are shown in **Figure** **5**. The portal also provides the data of PV panel's total output power (W), daily, monthly, and yearly energy (kWh) output and power–time graph which shows output power variation with day hours.

## 3 Optimal Tilt Angle Computation

The output of the PV panel is affected by the selection of inclination. Therefore, on the rooftop of UIET, Panjab University, Block‐I, 5 solar PV panel of rated capacity 1 kW each have been installed at different tilt angles. The determination of the optimal angle has been done in two steps as explained in the next sections.

### 3.1 Using Real Time Data

The real time data, from the month of January to December in the year 2018 logged in the portal, have been used to determine the best inclination of the panel. **Table** **4**a,b represents the data of total energy (in kWh) generation for different months at different angles in the year 2018.

**Table 4:** Energy generation (in kWh) for different angle for the year 2018

a						
Angle [degree]	Jan	Feb	Mar	Apr	May	Jun
10	42.4	42.7	76.1	57.9	68.2	95.2
20	49.1	63.9	79.4	57	73.1	93.7
25	51.1	67.7	86.8	60.9	77.2	97.4
30	53.5	68	85.1	57.1	68.675	86.8
40	76.6	66.9	80.4	56.5	65.9	84.5

b						
Angle [degree]	July	Aug	Sep	Oct	Nov	Dec
10	89.4	94.5	90.8	104.2	53.2	71.2
20	79.9	83.7	92.9	103.5	48.1	76
25	87.1	91	94.5	118.7	66.1	97.1
30	75.4	88	95.2	119.6	69.2	97.8
40	79.1	83	90.5	118.9	71	103.9

**Figure 6:**
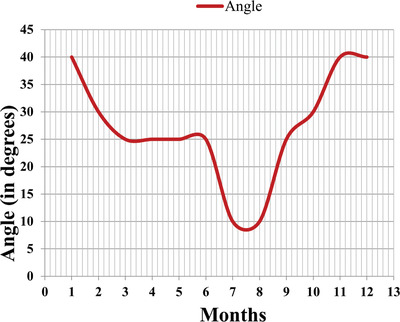
Monthly optimal tilt angle variation.

**Figure 7:**
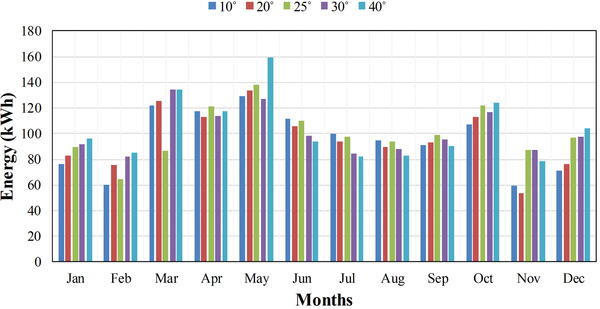
Month wise power output of PV panels at different tilt angles.

From Table 4a,b, the angles at which the maximum energy generation during different months have been observed and the thus optimal angles for specific months are tabulated in **Table** **5**. For example, in March month, energy at 25° angle which is 86.8 kWh had greater than the energy obtained at other inclination angles. Therefore, it is the optimal angle for March. Similarly, in August, 10° angle gives 94.5 kWh which had been greatest, so 10° is best angle for August month. In **Figure** **6**, the variation of optimal tilt angle with months has been shown. **Figure** **7** shows the monthly power output of each panel with respect to different angles. It is clearly shown from the graphs and tabular results that tilt angle becomes smaller during summer months when the sun is aligned vertically to the panel in the sky and becomes greater when winter comes.

**Table 5:** Monthly optimal tilt angle calculations for panel

Months duration	Optimal angle [degree]
November to January	40
February	30
March to June	25
July to August	10
September	25
October	30

### 3.2 Using Regression Analysis

An optimal angle enhances the energy output of the panels. Hence, the panels used for measurement are at five different angles in this study and it may be possible that higher generation can be obtained if angles are varied beyond their fixed values. This is not feasible practically to adjust the panel at every tilt angle and it also increases the cost and works in the installation process of the panel. So, the regression analysis method has been used in the study to analyze the energy output from various angles.

Regression is a basic and commonly used type of predictive analysis. These regression estimates are used to find the relationship between one dependent and one or more independent variables. The simplest form of regression is of the type *y* = *mx* + *c*, where *y* is the dependent variable, *m* is the slope, *x* is independent variable and *c* are constant. Polynomial regression in which degree of the independent variable is more than 1 is also used to determine predictive analysis such as time series, stock market share price, etc.[qv: 21]

**Figure 8:**
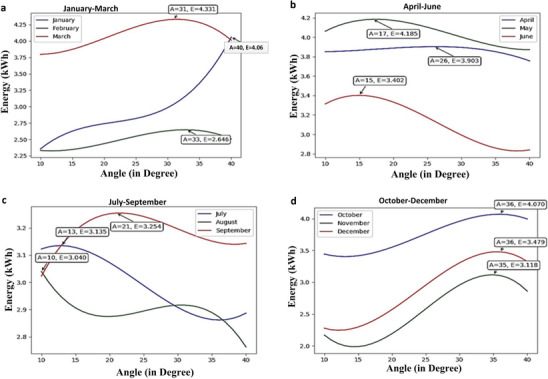
Monthly optimal tilt angle using regression analysis.

**Figure 9:**
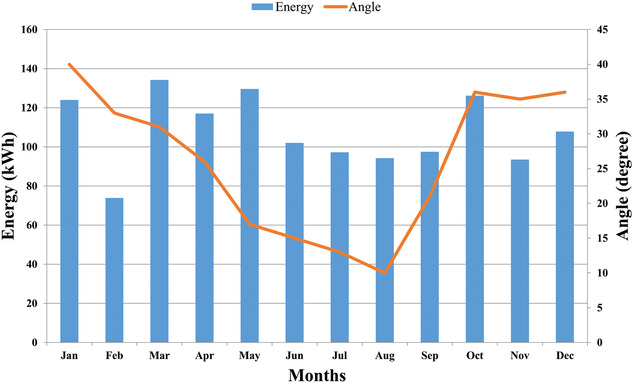
Graphical representation of energy generated with optimal tilt angle.

In the present work, the real time data collected from the Solax Web Portal for installed panels have been used for the analysis. Dataset so collected contains the attribute “angle” as the independent variable and “energy” as the dependent variable. The polynomial regression on this data has been applied in which the degree of the independent variable is 3. This gives the month‐wise relationship between energy and tilt angle which has been shown graphically for each month in **Figure** **8**. Wherein Figure 8a, the variation of energy (kWh) with tilt angles for January, February, and March and maxima point is depicted on each curve which represents tilt angle (A) corresponding to maximum average energy per day (E) for that particular month. The maxima point of March curve shows tilt angle (A) that is 31° at which average energy generated (E) per day is 4.33 kWh. It has been observed from the graphs that in summer season optimal tilt angle obtained for maximum generation is smaller as compared to the winter season. This variation in the tilt angle matches the results of the tilt angle obtained from real data. By using regression analysis, monthly energy obtained at the optimal tilt angle has been calculated as shown in **Table** **6**. The results obtained from regression analysis reveal that the tilt angle of the panel decreases first from January to August and then increases from September to December. The variation of the tilt angle so obtained is similar to the experimental result and also the variation of energy with monthly optimal tilt angle has been presented in **Figure** **9**.

**Table 6:** Monthly energy output variation of PV panel and optimal tilt angle

Months	Optimal tilt angle [degree]	Energy [kWh]
Jan	40	124
Feb	33	73.92
March	31	134.23
April	26	117
May	17	129.58
June	15	102.06
July	13	97.18
Aug	10	94.24
Sept	21	97.5
Oct	36	126.17
Nov	35	93.54
Dec	36	107.84

## 4 Result and Discussions

**Figure 10:**
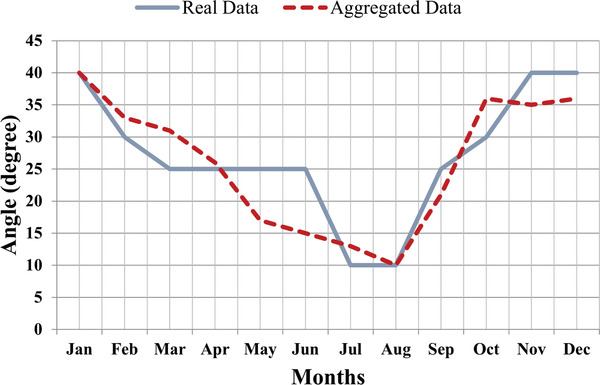
Graphical comparison of real and aggregates angles.

The annual optimal tilt angle for the Chandigarh region varies approximately from 26° to 28° as obtained from both experimental and simulation studies. Further, the comparison has been made between the real time data study and simulation results. It has been seen that the result trends remain almost similar for the real time and aggregated optimal angle that has been shown in **Table** **7** and graphically in **Figure** **10**.

**Table 7:** Monthly optimal tilt angle from realistic data and simulation method

Months	Real data angles [degree]	Regression result [degree]
Jan	40	40
Feb	30	33
March	25	31
April	25	26
May	25	17
June	25	15
July	10	13
Aug	10	10
Sept	25	21
Oct	30	36
Nov	40	35
Dec	40	36

### 4.1 Comparative Study between Real Time and Regression Data

The real time data has been recorded for the PV panels at fixed angles and then optimal angles have been determined. Regression method simulation results are used for the validation of these real time data experimental results as discussed in the next sections.

#### 4.1.1 Optimal Tilt Angle

The monthly optimal angle varies between 10° and 40° throughout the year as shown in Table 7. When the panel becomes perpendicular to the incident irradiance of the sun, it receives maximum irradiance. During summer months, that is, from May to July, the sun is on Tropic of Cancer (23.5° north) and positioned high in the sky at meridian, therefore optimal angle attains low values to be at 90° angle with irradiance and during winter months, that is, from November to January, sun reaches on Tropic of Capricorn (23.5° south) and it reaches maximum 35°, therefore tilt angles at that time is on the higher side (between 35° and 40°).

#### 4.1.2 Optimal Output Energy

**Figure 11:**
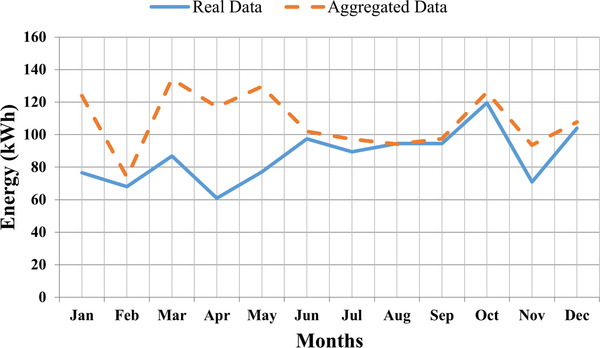
Graphical representation of real time and aggregated energy.

The energy generated from the solar PV experimental setup and calculated through regression analysis for each month have been shown in **Table** **8**. The results show little variation in real and aggregated data energy. This is due to the fact that for real time study, only five discrete angles (10°, 20°, 25°, 30°, 40°) have been considered for analysis, however, regression study takes the continuous angle between the range of 10° to 40° for energy calculation for a year. **Figure** **11** shows the variation in energy for each month. The graphical plot shows that the pattern of energy generated through PV panels at the optimal angle and through regression analysis is almost the same. Hence, the effectiveness of the proposed technique has been validated.

**Table 8:** Real time energy versus aggregated data energy

Months	Real data energy [kWh]	Aggregated data energy [kWh]
Jan	76.6	124
Feb	68	73.92
March	86.8	134.23
April	60.9	117
May	77.2	129.58
June	97.4	102.06
July	89.4	97.18
Aug	94.5	94.24
Sept	94.5	97.5
Oct	119.6	126.17
Nov	71	93.54
Dec	103.9	107.84

#### 4.1.3 Tilt Angle Effect on Generation and Carbon Offset Calculations

The angles recommended for installing any solar PV panel at the selected location are generally 30° and 40°. The effectiveness of the optimal angles suggested in the proposed study has been analyzed by comparing the energy response obtained at recommended fixed angles (30° and 40°) as represented in **Table** **9**.

**Table 9:** Panel output generation with optimal and fixed angle

Months	Optimal angle [degree]	Optimal angle energy [kWh]	Energy at 40° [kWh]	Energy at 30° [kWh]
Jan	40	76.6	76.6	53.5
Feb	30	68	66.9	68
Mar	25	86.8	80.4	85.1
Apr	25	60.9	56.5	57.1
May	25	77.2	65.9	68.675
Jun	25	97.4	84.5	86.8
Jul	10	89.4	79.1	75.4
Aug	10	94.5	83	88
Sep	25	99	90.5	95.2
Oct	30	119.6	118.9	119.6
Nov	40	71	71	69.2
Dec	40	103.9	103.9	97.8
	Total	5221.5	4886	4821.875

**Figure 12:**
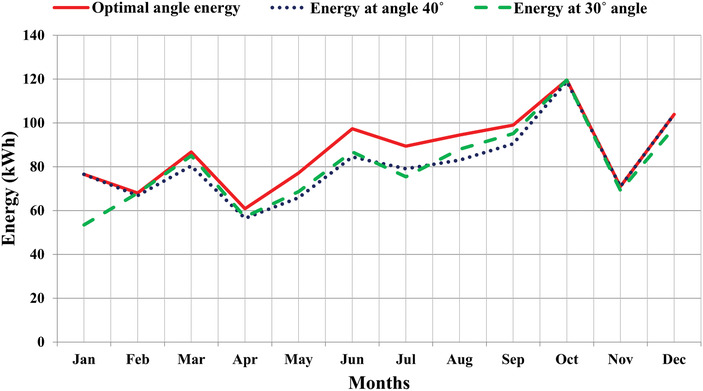
Graphical representation of optimal energy with fixed angle energy.

It has been shown that the energy generated throughout the year using optimal angles are higher as compared to the two fixed angles 30° and 40°, respectively. Also, graphically from **Figure** **12**, it has been seen that monthly optimal tilt angle energy generation is greater than the fixed angle energy in most of the time throughout the year. The comparative analysis concludes that optimally placed PV generates 6–7% more energy than the recommended angles of solar PV systems in the region of study.

**Figure 13:**
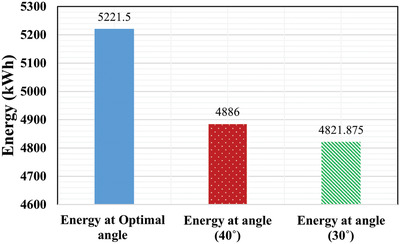
Optimal total energy versus fixed‐tilt angles energy.

It can be seen in **Table** **10** that the optimal energy obtained is 5221.5 kWh, whereas the energy generated at fixed angle of 40° is 4886 kWh which is approximately 336 kWh less than optimal angle energy, similarly for 30°, energy obtained is 4822 kWh having a difference of 400 kWh Therefore, if the PV panel is adjusted monthly at optimal tilt angle rather than fixed at 40° and 30° tilt angles, energy generation can be increased from 7% to 8% from the fixed angle energy. The same has been depicted graphically in **Figure** **13**.

**Table 10:** Panel output energy with optimal tilt angle and fixed angle

Angles [degree]	Energy Generated [kWh]	Angles [degree]
Optimal angle energy	5221.5	Optimal angle energy
Energy at 40°	4886	Energy at 40°
Energy at 30°	4821.875	Energy at 30°

The analysis reveals that the net savings using tilt angle optimization compared with fixed angles of 40° and 30° is approximately 336 and 400 kWh, respectively. According to the CEA report, the average CO_2_ emitted per unit of electricity generated in the grid through conventional generating sources is 0.82 TCO_2_/MWh.[qv: 22] Therefore, the reduction in CO_2_ emission when the generating is done in the PV system by placing at optimal angles throughout the year can be calculated as follows:

i) 336 kWh = 0.336 MWh = 0.336 × 0.82 = 0.27552, TCO_2_ = 27.552 kg CO_2_ emission
ii) 400 kWh = 0.4 MWh = 0.4 × 0.82 = 0.328, TCO_2_ = 32.8 kg CO_2_ emission

From the above calculation it is concluded that the proposed optimally placed solar panel of capacity 5 kW can help in reducing CO_2_ emission in the range of 27.55 kg to 32.8 kg in a year. If there is large solar PV plant installed with optimal tilt angles, then carbon offset savings are very large which will be beneficial from both economic and environmental points of view.

#### 4.1.4 Tilt Angle Adjustments Schedules

The optimal tilt angle of the panel changes for every month, therefore, it is required to have a tilt adjustment schedule on a seasonal basis so that adjustment frequency of angle will be minimized and the output energy of the panel is maximized. For that purpose, it has been proposed that there will be four tilt adjustments in a year after every 3 months interval starting from January and the optimal tilt for that duration has been calculated by finding the average of optimal tilt angle for 3 months. Tilt angles value for 3 months interval has been shown in **Table** **11** as follows:

**Table 11:** Tilt angles adjustments schedules

Months intervals	Tilt angle [degree] using real data	Using simulated data [degree]
Jan–March	31.67–32	34.66–35
April–June	25	20
July–Sept	15	15
Oct–Dec	36.67–37	36

**Figure 14:**
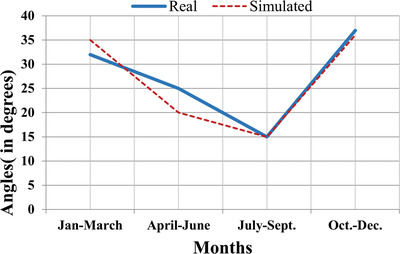
Optimal tilt angle adjustments on quarterly basis.

The optimal tilt angle variation for 3 months interval with real data and regression data is shown in **Figure** **14**. The results obtained through simulation and experimental setup follow a similar graphical pattern that validates the study.

## 5 Conclusion

In the present work, the study on the optimal tilt angle of the PV panel for the Chandigarh region has been done. It can be seen that the tilt angle for winter is greater than in summer due to the position of the sun in the sky. It has also been found that the annual tilt angle for the region varies approximately 26–28°. The experimental results have been compared with the result of regression analysis to find out the optimal tilt angle of the PV panel. It can be concluded that optimal tilt angle varies as a function of latitude, and also affected with solar radiation (kWh m^−2^) fall in the region. The optimal angles found after the experimental and theoretical analysis for the selected location are 32° (Jan–March), 25°(April–June), 15°(July–Sept), and 37°(Oct–Dec). The main advantage of the proposed angle setting of the panel is an increase in annual power output of the PV panel by 7–8% which helps to reduce the CO_2_ emissions_._ The same methodology will also be used in the other regions in India and other countries of the world for PV panel installation. This proposed approach is quite effective and feasible for implementing in large PV plants in India, therefore, increase the generation of plants significantly in economical way and help developing nations like India to cater to the need for electricity demand along with economic benefits.

## Conflict of Interest

The authors declare no conflict of interest.
